# Health care reform in the US: an analysis of implementation of the Affordable Care Act in California

**DOI:** 10.1186/1472-6963-14-S2-P103

**Published:** 2014-07-07

**Authors:** Dylan Roby, Ken Jacobs, Greg Watson, Alla Bronshteyn, Dave Graham-Squire, Michelle Keller

**Affiliations:** 1 UCLA Center for Health Policy Research, Los Angeles, CA, USA; 2 Department of Health Policy and Management, UCLA Fielding School of Public Health, Los Angeles, CA, USA; 3 UC Berkeley Center for Labor Research and Education, Berkeley, CA, USA; 4 Department of Biostatistics, UCLA Fielding School of Public Health, Los Angeles, CA, USA

## Background

The Patient Protection and Affordable Care Act (ACA) was signed into law in 2010. In the past four years, federal and state governments have made changes to existing programs, created new ones, and attempted to foster innovation in the health care system. We provide an update on implementation of the ACA in California, specifically focused on the expansion of Medi-Cal for low-income populations and the creation of California’s marketplace to purchase insurance coverage.

## Materials and methods

The main analysis is based upon a microsimulation model created by UCLA and UC Berkeley to predict the impact of the ACA on California, given the unique demographic differences in California and the varied implementation of the law in the state. This model is built using economic and probability theory, coupled with multiple data sets on the health care use of Californians (Medical Expenditure Panel Survey), the behavior of employers (Employment Development Department and the California Employer Health Benefits Survey), and characteristics of California’s population (the California Health Interview Survey). This model provides a baseline picture of insurance coverage in California without the ACA, and also allows us to estimate the impact of the ACA and compare it to the actual data released on insurance status, take-up, and response in 2014.

## Results

Taking into account churn, which is predicted to reduce enrolment by more than 40% on an annual basis [1], between 1.1 and 1.3 million people will be enrolled in Covered California with subsidies at any point in time. Enrolment in the individual market or Covered California without subsidies will range from 2.4 to 2.7 million. Medi-Cal enrolment will reach an all-time high ranging from 7.4 to 7.8 million.

## Conclusions

Changes to insurance regulations, the creation of health insurance marketplaces, and the expansion of Medicaid in California have reduced the number of uninsured individuals substantially. However, the choices available to consumers, the requirement to purchase insurance, and the cost of insurance coverage could be difficult barriers to widespread acceptance of health reform in the U.S. and California.
